# Habitual carbohydrate intake is not correlated with circulating β-hydroxybutyrate levels in pregnant women with overweight and obesity at 28 weeks' gestation

**DOI:** 10.1007/s00125-023-06044-w

**Published:** 2023-11-16

**Authors:** Helen L. Tanner, Hui Ting Ng, Grace Murphy, Helen L. Barrett, Leonie K. Callaway, H. David McIntyre, Marloes Dekker Nitert

**Affiliations:** 1 Department of Obstetric Medicine, Royal Brisbane and Women’s Hospital, Metro North Hospital and Health Service, Herston, QLD Australia; 2 School of Chemistry and Molecular Biosciences, The University of Queensland, St Lucia, QLD Australia; 3 Department of Medicine, University of New South Wales, Sydney, NSW Australia; 4 Women’s and Newborn Services, Royal Brisbane and Women’s Hospital, Metro North Hospital and Health Service, Herston, QLD Australia; 5 Mater Clinical Unit, Faculty of Medicine, The University of Queensland, South Brisbane, QLD Australia

**Keywords:** Carbohydrate intake, Gestational diabetes mellitus, Glucose, Insulin, Ketones, Pregnancy

## Abstract

**Aims/hypothesis:**

Pregnant women are advised to consume a minimum of 175 g per day of carbohydrate to meet maternal and fetal brain glucose requirements. This recommendation comes from a theoretical calculation of carbohydrate requirements in pregnancy, rather than from clinical data. This study aimed to determine whether fasting maternal ketone levels are associated with habitual carbohydrate intake in a subset of participants of the Study of PRobiotics IN Gestational diabetes (SPRING) randomised controlled trial.

**Methods:**

Food frequency questionnaires on dietary intake during pregnancy were completed by pregnant women with overweight or obesity at 28 weeks’ gestation (considering their intake from the beginning of pregnancy). Dietary intake from early pregnancy through to 28 weeks was analysed for macronutrient intake. At the same time, overnight fasting serum samples were obtained and analysed for metabolic parameters including serum β-hydroxybutyrate, OGTTs, insulin and C-peptide.

**Results:**

Fasting serum β-hydroxybutyrate levels amongst 108 women (mean BMI 34.7 ± 6.3 kg/m^2^) ranged from 22.2 to 296.5 μmol/l. Median fasting β-hydroxybutyrate levels were not different between women with high (median [IQR] 68.4 [49.1–109.2 μmol/l]) and low (65.4 [43.6–138.0 μmol/l]) carbohydrate intake in pregnancy. Fasting β-hydroxybutyrate levels were not correlated with habitual carbohydrate intake (median 155 [126–189] g/day). The only metabolic parameter with which fasting β-hydroxybutyrate levels were correlated was 1 h venous plasma glucose (ρ=0.23, *p*=0.03) during a 75 g OGTT.

**Conclusions/interpretation:**

Fasting serum β-hydroxybutyrate levels are not associated with habitual carbohydrate intake at 28 weeks’ gestation in pregnant women with overweight and obesity.

**Graphical Abstract:**

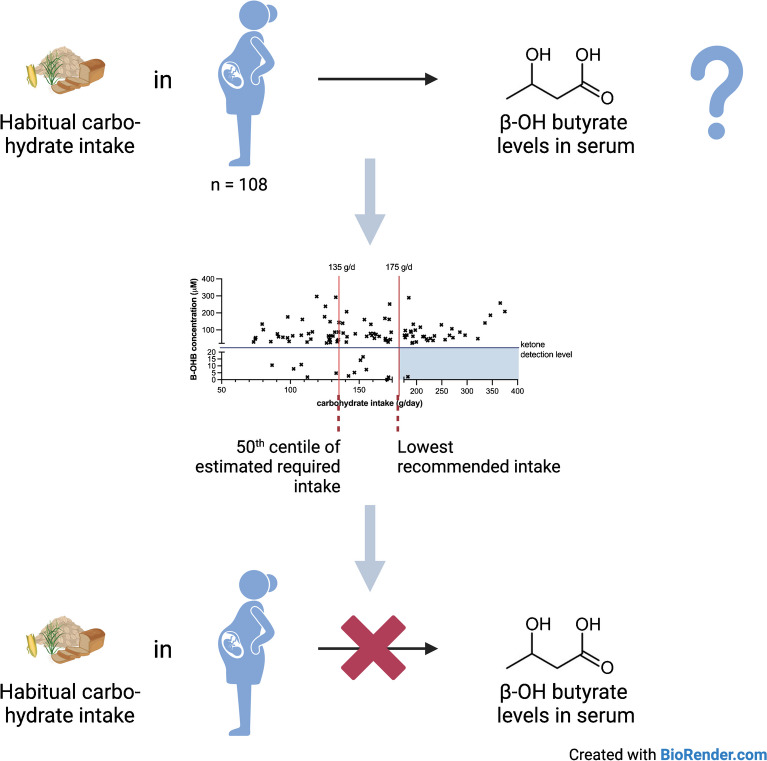

**Supplementary Information:**

The online version of this article (10.1007/s00125-023-06044-w) contains peer-reviewed but unedited supplementary material.


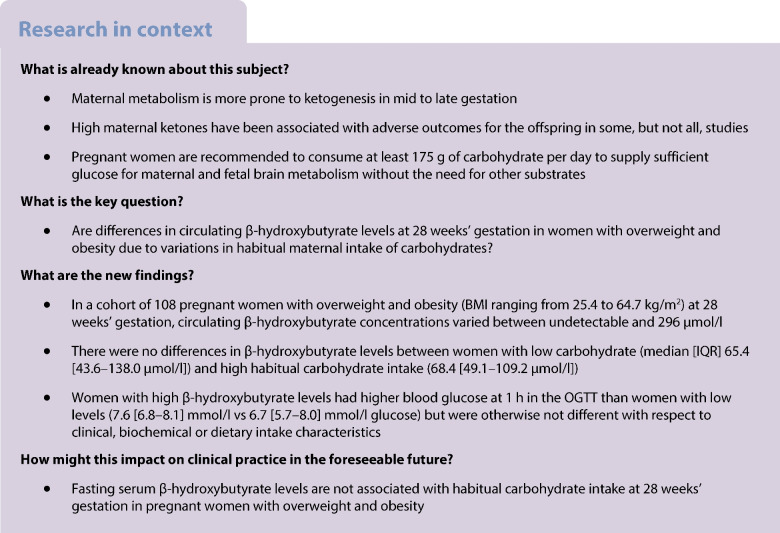


## Introduction

Current dietary advice for pregnant women is to avoid diets that lead to increased ketonaemia [1, 2]. This is owing to a concern about the effect of maternal ketones on the developing fetus. As part of this, it is recommended that pregnant women consume ≥175 g/day of carbohydrate [3]. This recommendation is based on dietary reference intakes published by the National Academies of Sciences in the USA, where the amount of carbohydrate that women need to consume to supply sufficient fuel to the brain of mother and fetus, without the need for other fuel substrates, and to avoid ketones, has been calculated at 175 g/day [4]. This is based on the calculation of the estimated mean intake of 135 g/day, of which 100 g is the calculated use by the average maternal brain and 33 g (rounded to 35 g) for use by an average size fetal brain [2]. In order to ensure that the needs of 97.5% of the pregnant population are covered, two times the population coefficient of variation of 15% of the 135 g/day is added to reach a recommended daily carbohydrate intake of 175 g/day [2]. A recent study additionally calculated that, in addition to maternal and fetal brain glucose consumption, placental glucose consumption should be taken into account, which would further increase the daily recommended carbohydrate intake to 220 g/day [5]. In addition, ketone body production increases rapidly after 12–14 h of fasting in pregnant women, along with rapid rises in circulating NEFA levels and decreasing glucose levels, whereas in non-pregnant women this occurs only after 18 h of fasting [6]. There are, however, no clinical studies showing that routine consumption of less than 175 g/day of carbohydrate leads to increased ketone production in pregnant women. A study of a single week of low energy intake in women with obesity (5020 kJ/day achieved by reductions in portion size, not number of meals or type of food, and carbohydrate intake of ∼150 g/day) showed a significant increase in the fasting levels of the ketone β-hydroxybutyrate (B-OHB) at the end of the exposure [7]. The concern regarding maternal ketones stems from older studies showing an association between maternal ketones and adverse fetal and childhood outcomes, particularly reduced childhood intelligence quotient (IQ) [8]. However, later studies evaluating this association had conflicting results or did not account for potential confounding factors known to affect childhood IQ, thereby making it difficult to interpret the validity of the findings [9].

Ketones are produced from the breakdown of lipids when glucose availability is low. Ketogenesis is increased in pregnancy, particularly in the third trimester [6]. The main ketone body is B-OHB and normal levels are considered to be less than 500 μmol/l (0.5 mmol/l). However, maternal serum B-OHB levels ranging from as low as 0.07–0.25 mmol/l (or 70–250 μmol/l) have been found to be inversely associated with childhood IQ, although with a low correlation coefficient (*r*=−0.20) and in a cohort of 223 women of whom 89 had pre-existing and 99 had gestational diabetes mellitus (GDM) and whose carbohydrate intake was not reported [10]. While glucose availability is thought to be the main determinant of ketone levels, studies have shown that other factors, such as overall energy restriction [7] and the maternal microbiome [11], are associated with maternal ketone levels.

Outside pregnancy, a diet with less than 50 g of carbohydrate per day is generally considered to be ketogenic [12]. The level of carbohydrate restriction that leads to increased ketone production in pregnancy remains to be determined. Studies evaluating maternal ketone levels and their relationship to dietary intake are scarce. One trial of a low-carbohydrate diet in women with GDM randomised to 135 g/day carbohydrate did not result in increased fasting B-OHB levels, which had a mean of around 100 μmol/l, and which could be due to their actual consumption of 165 g/day carbohydrate rather than the intended 135 g/day [13]. The Diabetes and Pregnancy Vitamin D and Lifestyle Intervention for Gestational Diabetes Mellitus Prevention (DALI) study also measured fasting B-OHB levels, which were between 65 and 82 μmol/l at the start of the third trimester of pregnancy and were not affected by physical activity but were increased in the group assigned to a healthy-eating diet [14]. In the DALI cohort, there was a weak negative correlation between B-OHB levels and the total amount of carbohydrate consumed, determined as the product of times per week and portions per occasion [14]. At present, there is limited information available to clinicians to advise pregnant women about the likelihood of elevated ketone levels with consumption of a low-carbohydrate diet.

In order for clinicians to be able to provide women with accurate advice about carbohydrate intake in pregnancy, we need to better understand the relationship between maternal dietary carbohydrate intake and serum B-OHB levels. To our knowledge there are no studies that analyse fasting B-OHB levels in pregnant women and correlate these levels with carbohydrate intake. Our hypothesis was that low habitual carbohydrate intake is associated with higher circulating B-OHB concentrations. The aim of this study was to determine the association between daily habitual carbohydrate intake from early pregnancy through to 28 weeks’ gestation and serum B-OHB levels at 28 weeks’ gestation in pregnant women with overweight and obesity.

## Methods

Food frequency questionnaires were completed in a subset of 108 women with uncomplicated pregnancies enrolled in the Study of Probiotics IN Gestational Diabetes (SPRING), an RCT of probiotics to prevent GDM in pregnant women with overweight and obesity (ACTRN12611001208998) [15]. All women provided written informed consent to participate in the study and the study was approved by the Human Research Ethics Committees of the Royal Brisbane & Women’s Hospital (11/467) and the University of Queensland (2012000080). Dietary intake was assessed at 28 weeks’ gestation using the validated Cancer Council Victoria’s Dietary Questionnaire for Epidemiological Studies V2.0 [16, 17] and women were asked to complete the questionnaire considering their intake from the beginning of pregnancy through to 28 weeks’ gestation (referred to throughout as their ‘habitual carbohydrate intake’). Dietary intake was analysed for total daily energy (in kJ), carbohydrate, fat, protein, sugar, starch and fibre intake. The dietary data were obtained at the same study visit as the demographic data and the fasting serum samples.

Demographic data were collected for each woman including ethnicity, maternal age at delivery, maternal BMI at the 28 week-gestation antenatal visit, and intervention group in the study (probiotic or placebo). The study population by design has a higher BMI than the general obstetric population in Australia; it has a higher proportion of women with white ethnicity and who have completed tertiary education (as a proxy for socioeconomic status) [15]. Fasting serum samples were taken from women at the 28 week-gestation antenatal visit and metabolic parameters were obtained including an OGTT, fasting insulin, fasting C-peptide and fasting lipids. Insulin resistance (HOMA-IR) was calculated using fasting blood glucose and insulin levels (HOMA-IR = insulin × glucose/22.5). Insulin secretion (HOMA-B) was calculated using fasting insulin and glucose levels (HOMA-B = 20 × insulin/[glucose – 3.5]). Women were asked to fast for 9.5–12 h prior to their OGTT.

Mass spectrometry was used to analyse serum B-OHB levels. Analytical reagents DL-β-hydroxybutyric acid sodium salt (β-HBA, catalogue no. H6501), 3-nitrophenyl hydrazine (3NPH, catalogue no. N21804), *N*-(3-dimethylaminopropyl)-*N*′-ethyl carbodiimide (EDC, catalogue no. 39391), 2-ethyl butyric acid (2EBA, catalogue no. 109959), pyridine (catalogue no. 270407) and acetonitrile (ACN, catalogue no. 34851) were purchased from Sigma-Aldrich (Macquarie Park, NSW, Australia). A 10 mmol/l β-HBA stock solution was prepared using Milli-Q water and diluted using 50% ACN (vol./vol.) to create 20–400 µmol/l standards. A 5 µmol/l internal standard was prepared using 2EBA in 100% ACN. Ten microlitres of standards or serum samples were transferred to an Eppendorf tube and combined with 100 µl of 5 µmol/l 2EBA solution. The mixture was centrifuged at 12,000 *g* for 10 min at 4°C. A 90 µl aliquot of supernatant was transferred to a glass HPLC vial with 45 µl of 200 mmol/l 3NPH in 30% ACN and 45 µl of 120 mmol/l EDC in 6% pyridine solution in 100% ACN (vol./vol.). The mixture was incubated for 30 min at 40°C, then 1.8 ml of 10% ACN was added to complete derivatisation and samples were refrigerated until analysis.

Ultraperformance liquid chromatography-tandem mass spectrometry (UPLC-MS/MS) analysis was performed using a Shimadzu Nexera ultraperformance LC system (Kyoto, Japan) attached to a SCIEX 5500 QTRAP mass spectrometer (Framingham, MA, USA). Chromatographic separations were carried out using a Kinetex 2.6 μm 150×2.1 mm C18 column (Phenomenex, Lane Cove, NSW, Australia), with a total flow of 0.3500 ml/min at 40°C. The pressure range was 0–12,000 psi for Pump A/B and 0–900 psi for Pump C. Buffers for mobile phase for gradient elution consisted of Buffer A, made up of (vol./vol.) 1% ACN, 0.1% formic acid and Buffer B composed of 90% ACN, 0.1% formic acid. The mass spectrometer, coupled with an electron spray ionisation source, was operated in the negative multiple reaction monitoring (MRM) mode and included source parameters of ion spray voltage (ISV) at −4200 V; temperature 450°C; GS1 (nebuliser gas) 35; curtain gas 20. Each standard and sample was injected at a volume of 10 μl and subsequently 50 μl. Optimised parameters through manual infusion for maximum sensitivity for each compound transition included the collision energy, declustering potential and collision cell exit potential. Individual fragment ions were detected using the Q3 mass analyser to identify the Q1/Q3 pairs for each analyte. The two most sensitive pairs of Q1/Q3 per analyte were used for the subsequent analyses. The retention time for propionic acid was ~5.94, butyric acid was ~7.71, acetic acid was ∼4.17, β-HBA was ~4.10 and 2EBA was ∼10.14 min. UPLC/MRM-MS data was processed using Multiquant 3.0 (SCIEX) software to optimise Gaussian smoothing to peaks and retrieve area measurements.

Obstetric outcomes were obtained for each woman including GDM status defined according to the International Association of the Diabetes and Pregnancy Study Groups (IADPSG) guidelines [18], hypertensive disorders of pregnancy (HDP), gestational weight gain, infant birthweight and infant sex.

### Statistics

Women were divided into two groups; high carbohydrate intake (HC) as defined by carbohydrate intake greater than the median amount in g/day, and low carbohydrate intake (LC) defined as less than the median amount in g/day (155 g/day). Participant clinical, dietary and biochemical parameters are presented as mean ± SD (continuous data) or % (categorical data) and compared using two-sided *t* tests and χ^2^ tests. The data for B-OHB levels were not normally distributed and data are presented as median with IQR. Group comparisons for B-OHB levels were conducted using Mann–Whitney *U* tests. Correlations between B-OHB levels were conducted using Spearman’s correlation coefficient. A *p* value <0.05 was used as a cut-off for statistical significance.

## Results

### Participant characteristics

The cohort of 108 participants (mean BMI 34.7 ± 6.3 kg/m^2^ [range 25.4–64.7 kg/m^2^], median carbohydrate intake 155 [126–189] g/day) was split into one group of women with a lower habitual carbohydrate intake (LC, *n*=53) and another with higher habitual carbohydrate intake (HC, *n*=55). The individuals in the groups were matched for maternal BMI (± 5 kg/m^2^), maternal age (± 5 years), treatment arm allocation in the SPRING trial, ethnicity and infant sex. There were no significant differences between the groups in any of the demographic characteristics of the participants (Table 1). Two participants in the LC group and five in the HC group were diagnosed with GDM at 28 weeks’ gestation and seven participants in the LC and four participants in the HC group developed HDP in late pregnancy (Table 1).

**Table 1:** Participant demographics at 28 weeks’ gestation

	LC group	HC group	*p* value
*n*	53	55	ND
Maternal age	31.1±5.0	31.6±4.3	0.60
White ethnicity *n* (%)	49 (92.5)	52 (94.5)	0.71
Study allocation Probiotics/Placebo (*n*)	28/25	29/26	0.99
Parity <1 (*n*)	12	8	0.33
Maternal BMI at 28 weeks (kg/m^2^)	34.9 ± 5.4	34.4 ± 6.6	0.68
SBP (mmHg)	111 ± 10	109±9	0.38
DBP (mmHg)	66 ± 7.6	66±7.8	0.93
OGTT-Fasting glucose (mmol/l)	4.3±0.3	4.2±0.3	0.63
OGTT-1 h glucose (mmol/l)	7.3±1.4	7.2±1.6	0.52
OGTT-2 h glucose (mmol/l)	6.1±1.3	6.2±1.4	0.72
Fasting insulin (pmol/l)	63.8±32.9	54.1±26.2	0.09
Fasting cholesterol (mmol/l)	6.6±1.1	6.9±1.3	0.19
Fasting HDL-cholesterol (mmol/l)	1.8±0.4	1.9±0.4	0.31
Fasting LDL-cholesterol (mmol/l)	3.8±1.0	4.0±1.2	0.26
Fasting VLDL-cholesterol (mmol/l)	1.0 ± 0.3	1.0 ± 0.3	0.89
Fasting triglycerides	2.2±0.8	2.1±0.7	0.80
GDM (*n*)	2	5	0.44
HDP (*n*)	7	4	0.36
GWG (kg)	8.7±6.4	8.6±5.2	0.88
GA at delivery (weeks)	39.8±1.1	39.9±1.3	0.93
Infant birthweight (g)	3732±458	3647±491	0.36
Infant sex: Female/Male (*n*)	28/25	26/29	0.70
Infant length (cm)	52.1±2.8	52.5±2.5	0.52
Infant head circumference (cm)	35.3±1.5	35.3±1.6	0.91
SGA (<10th centile) *n*	1	0	0.99
LGA (>90th centile) *n*	10	7	0.59

Data are presented as mean ± SD

DBP, diastolic blood pressure; GA, gestational age; GWG, gestational weight gain (across gestation); *n,* number of participants; ND, not determined; SBP, systematic blood pressure; SGA, small for gestational age

The routine dietary intake from the beginning of pregnancy until 28 weeks’ gestation as measured by food frequency questionnaire was different for overall energy intake, portion of food consumed and all macronutrients with the participants in the HC group consuming higher amounts (Table 2). As an indication of dietary composition, we also compared the proportions of the macronutrients normalised for energy intake between the groups. The proportions were only different for carbohydrate intake as per grouping, which was higher in the HC group (LC 24.3±2.6 vs HC 25.5±2.8 mg/kJ, *p*=0.02). By contrast, total fat intake was lower in the HC group (LC 10.7±1.0 vs HC 10.2±1.1 mg/kJ, *p*=0.007) as was saturated fat intake (LC 4.7±4.5 vs HC 4.4±0.6 mg/kJ, *p*=0.01) (ESM Table 1).

**Table 2:** Dietary intake in pregnancy until 28 weeks’ gestation

	Full cohort	LC group	HC group	*p* value^a^
*n*	108	53	55	ND
Overall energy intake (kJ/day)	6582±2279	4928±1029	8175±2001	<0.0001
Carbohydrate (g/day)	163.5±57.8	118.6±22.4	206.7±47.5	<0.0001
Sugars (g/day)	76.8±29.1	55.6±12.4	97.3±25.6	<0.0001
Starch (g/day)	85.9±34.1	62.5±17.6	108.4±30.9	<0.0001
Fibre (g/day)	18.2±6.9	13.4±3.4	22.9±6.2	<0.0001
Protein (g/day)	76.8±28.8	57.7±15.2	95.2±26.9	<0.0001
Total fat (g/day)	68.8±25.5	53.1±13.5	83.9±25.3	<0.0001
Saturated fat (g/day)	30.0±11.3	23.6±6.9	36.2±11.3	<0.0001
Polyunsaturated fat (g/day)	8.5±3.8	6.6±2.3	10.4±4.0	<0.0001
Monounsaturated fat (g/day)	24.3±9.6	18.4±5.1	29.9±9.5	<0.0001

Data are presented as mean ± SD

*n*, number of participants; ND, not determined

^a^*p* value comparison between LC and HC groups

### Circulating B-OHB levels

**Fig. 1:**
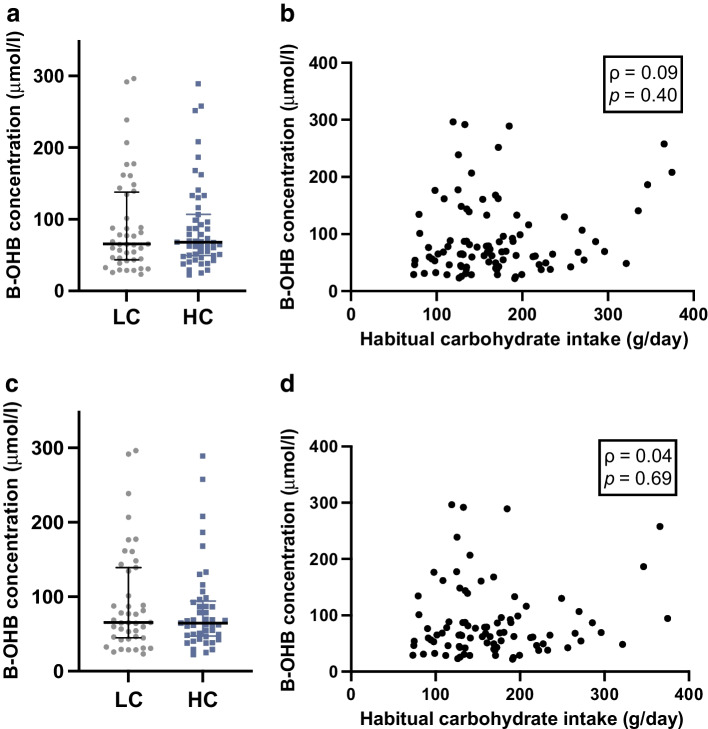
B-OHB concentrations and carbohydrate intake. (**a**) B-OHB concentrations in the LC and HC groups. (**b**) Spearman correlation between habitual carbohydrate intake and circulating B-OHB concentrations. (**c**) B-OHB concentrations in normoglycaemic LC and HC participants. (**d**) as in (**b**) but only including normoglycaemic participants. Data presented as individual points, and with median and IQR in (**a**) and (**c**)

The limit of detection for B-OHB for our measurement was 20 μmol/l and the range in the overall cohort was from 22.2 to 296.5 μmol/l. In the LC group, nine participants (17.0%) did not show detectable levels of B-OHB in their fasting blood. In the HC group, four participants (7.3%) had no detectable B-OHB levels (*p*=0.15). These 13 participants, who included one participant with GDM, were excluded from the analyses. The median (IQR) B-OHB concentrations were similar in participants with low and high carbohydrate intake: LC 65.4 (43.6–138.0 μmol/l) vs HC 68.4 (49.1–109.2 μmol/l), *p*=0.87 (Fig. 1a). Routine carbohydrate intake was not correlated with circulating B-OHB concentrations at 28 weeks’ gestation (ρ=0.09, *p*=0.40; Fig. 1b). In this cohort, there were seven participants with GDM. The presence of GDM could affect the synthesis of ketone levels and therefore the analysis was repeated in normoglycaemic participants only. The median (IQR) of B-OHB in the LC group was 65.5 (44.8–139.2) μmol/l vs 64.7 (47.4–94.5) μmol/l in the HC group, *p*=0.72) (Fig. 1c). Similarly, when including only normoglycaemic participants, there was still no correlation between routine carbohydrate intake and B-OHB levels (ρ=0.04, *p*=0.69) (Fig. 1d). These results indicate that variability in B-OHB levels is not explained by routine carbohydrate intake. The data were also analysed to determine whether consumption of more or less carbohydrate than the recommended guideline of 175 g/day was associated with B-OHB levels. The median (IQR) B-OHB concentrations were similar in the 33 participants without GDM consuming ≥175 g/day (68.8 [48.9–111.5] μmol/l B-OHB) compared with the 66 normoglycaemic participants consuming <175 g/day carbohydrate (65.5 [44.1–133.8] μmol/l B-OHB, *p*=0.75).

**Fig. 2:**
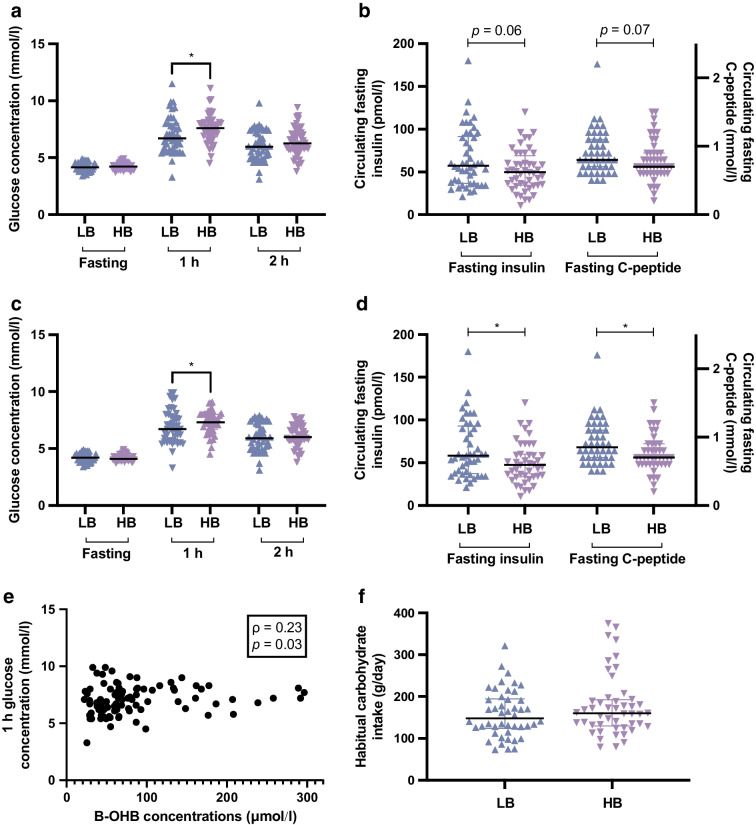
Differences in glucose and insulin between participants in the LB and HB groups. (**a**) Fasting, 1 h and 2 h glucose levels in the OGTT at 28 weeks’ gestation in participants in the LB and HB groups. (**b**) Fasting insulin and C-peptide levels before the OGTT at 28 weeks’ gestation in the LB and HB groups. (**c**, **d**) As in (**a**, **b**) but only including normoglycaemic participants. (**e**) Spearman correlation between B-OHB concentrations and glucose concentrations at 1 h in the OGTT. (**f**) Habitual carbohydrate intake between women in the LB and HB groups. Data presented as individual points, and with a line through the median in (**a**–**d**), *p<0.05

Given that the B-OHB concentrations at 28 weeks’ gestation were quite variable, we therefore investigated whether dietary intakes of other macronutrients were different between participants with B-OHB concentrations above and below the median of 65 μmol/l. There were no significant differences between the low (LB) and high (HB) B-OHB groups in routine dietary macronutrient intake, maternal BMI, age, blood pressure, gestational weight gain, infant birthweight or length, or in circulating maternal cholesterol or triglycerides (ESM Tables 2 and 3). However, the participants with higher B-OHB concentrations (HB) had higher blood glucose levels at 1 h in the OGTT: HB group median (IQR) 7.6 (6.8–8.1) mmol/l glucose vs LB group 6.7 (5.7–8.0) mmol/l glucose, *p*=0.016 (Fig. 2a, ESM Table 2). There were no differences in fasting glucose or glucose at 2 h in the OGTT (Fig. 2a). The fasting insulin and C-peptide levels were borderline lower in the HB group vs LB group (*p*=0.06 for insulin and *p*=0.07 for C-peptide) (Fig. 2b, ESM Table 2). The HOMA-IR values were borderline lower in the HB group (1.5 [1.0–2.1]) than in the LB group (1.8 [1.1–2.9]) (*p*=0.07). Given that there were a number of participants with GDM in this group, we repeated the analysis without these participants. The results were similar, with the participants in the HB group again having higher circulating glucose at 1 h in the OGTT: HB group 7.3 (6.7–8.0) mmol/l glucose vs LB group 6.7 (5.7–7.9) mmol/l glucose, *p*=0.04) and significantly lower fasting insulin (*p*=0.02) and C-peptide (*p*=0.02) levels and no differences in fasting or 2 h glucose (Fig. 2c,d). HOMA-IR was now significantly lower in the HB group (1.5 [1.0–2.0]) compared with the LB group (1.8 [1.1–2.9]) (*p*=0.04). Of these factors, only the 1 h OGTT glucose was significantly correlated with circulating B-OHB levels (ρ=0.23, *p*=0.03) (Fig. 2e). Dietary carbohydrate intake was, however, not correlated with 1 h OGTT glucose levels (ρ= −0.02, *p*=0.86). ESM Tables 4 and 5 show the correlations between fasting B-OHB levels and other maternal and infant characteristics and dietary intake. To ensure that the results do not reflect an insufficient separation of the B-OHB concentrations, we divided the cohort into quartiles and analysed the same maternal characteristics and dietary intake data between Q1 (median B-OHB 35 μmol/l, IQR 29–42 μmol/l) and Q4 (median B-OHB 165 μmol/l, IQR 140–231 μmol/l). Again, only the 1 h blood glucose levels in the OGTT were significantly higher in Q4 (Q1 6.7 [5.7–7.8], Q4 7.9 [7.0–8.3] mmol/l, *p*=0.01); none of the other characteristics were significantly different (data not shown).

## Discussion

In this cohort of women with overweight and obesity at 28 weeks’ gestation of pregnancy, with a median habitual dietary carbohydrate intake of 155 g/day, maternal fasting serum B-OHB levels ranged from 22.2 to 296.5 μmol/l (0.0222–0.297 mmol/l). While this is below 500 μmol/l or 0.5 mmol/l, which is the level considered to be normal, it is within the range of levels that has previously been associated with reduced childhood IQ (70–250 μmol/l or 0.07–0.25 mmol/l) [10]. The levels in this study were, therefore, relevant to previous concerns about adverse childhood outcomes.

We found that in pregnant women with overweight and obesity, routine daily carbohydrate consumption from early pregnancy through to 28 weeks’ gestation was not correlated with fasting serum B-OHB levels. This finding remained after exclusion of women with GDM, suggesting the lack of correlation is not related to women’s carbohydrate tolerance. In addition, women with habitual low carbohydrate intake did not have higher serum B-OHB levels than those with high carbohydrate intake. This is despite the mean carbohydrate intake in the LC group being 119 g/day and hence 30% below the recommendation of ≥175 g/day, and the mean carbohydrate intake in the HC group being 207 g/day and 18% above the recommended minimum intake. The variability in fasting serum B-OHB levels cannot therefore be explained by habitual carbohydrate intake.

Amongst women with normoglycaemic pregnancies, those with higher serum B-OHB levels had lower fasting insulin and C-peptide levels. One explanation for this could be the inhibitory effect that insulin has on ketone production. The production of ketones involves two processes: first lipolysis, where lipids (mostly from adipose tissue stores) are broken down into fatty acids and glycerol, and second ketogenesis, where fatty acids are converted to ketones in the liver. Lipolysis is inhibited by insulin through inhibition of intracellular hormone-sensitive lipase [19] indicating that if insulin levels are high, fewer lipids are hydrolysed into fatty acids and glycerol and therefore ketone production is reduced. Conversely if insulin levels are lower, there is less inhibition of hormone-sensitive lipase, leading to higher triglyceride hydrolysis into fatty acids yielding more substrates for ketone production. Our finding of lower fasting insulin levels in women with higher serum B-OHB levels is therefore expected. We also found that women with higher serum B-OHB levels had higher glucose concentrations at 1 h in the OGTT. A potential explanation for this is that B-OHB levels were inversely correlated with fasting insulin levels. Lower fasting insulin levels may result in higher blood glucose levels at 1 h as there is insufficient insulin to lower the glucose [20]. This suggests that women with higher B-OHB levels secreted less insulin in response to the glucose load. Unfortunately, we do not have any measures of 1 h insulin concentrations to confirm this hypothesis.

This is the first study to evaluate the relationship between maternal ketone levels and habitual daily carbohydrate intake. Most previous studies looking at the effects of low-carbohydrate diets in pregnancy have been trials evaluating the effectiveness of carbohydrate restriction for glucose management in women with GDM. In one study, women on a low-carbohydrate diet (carbohydrate intake comprising less than 42% of total daily energy) had lower need for pharmacological treatment of their GDM and a lower incidence of large for gestational age (LGA) infants than women on a high-carbohydrate diet (carbohydrate intake greater than 45% of total daily energy) [21]. However, two of the women in the low-carbohydrate diet developed ketonuria, compared with none in the high-carbohydrate group, which was resolved by increasing carbohydrate intake [21]. Ketonuria was detected using the dipstick method in which the limit of detection of acetoacetate in the urine is around 0.5 mmol/l, which is significantly higher than the circulating B-OHB levels detected in our cohort of mostly normoglycaemic women. Given that circulating ketone levels were not measured in this study, it is not clear whether carbohydrate intake was related to lower levels of ketones in these women. In another study in which women were randomised either to consume 135 g/day of carbohydrate or to routine care (approximately 200 g/day carbohydrate), but where the low-carbohydrate group actually consumed 165 g/day of carbohydrate, there was no difference in fasting serum ketone concentrations between the two groups of women [13]. In the DALI study, circulating B-OHB was also measured at 24–28 weeks and levels were very similar to those we report here [14]. The DALI study did not have information on carbohydrate intake in g/day but rather as a product of frequency and number of portions based on a 12-item self-reported questionnaire, which they reported was weakly negatively correlated with B-OHB levels (*r* = −0.13, *p*<0.02). It is not clear how much carbohydrate this amounts to but the magnitude of the correlation suggests, especially when taking into account the findings of our and other previous studies, that there may not be a strong correlation between daily carbohydrate intake and fasting serum ketone levels in pregnancy.

Limitations of this study include the fact that dietary data was self-reported in a questionnaire, meaning there is a risk of recall bias. Given that the median weight gain in our cohort was 8 kg (4.7–12 kg) but the reported median energy intake is only 6136 kJ/day (4918–7489), it is very likely that the participants were underreporting their energy intake, as this is common in pregnant women with obesity [22]. Pregnant women who underreport their energy intake are more likely to report a higher % energy from carbohydrates [23]. However, given that there is no relationship between ketone levels and either gestational weight gain, energy intake or carbohydrates as % energy, we do not think that this underreporting impacts the lack of relationship between ketone levels and carbohydrate/energy intake. The food frequency questionnaire is also designed to obtain information about habitual dietary intakes, i.e. the average food intake across pregnancy in this study, rather than dietary intake in the 24 h prior to the fasting blood samples being taken. Potentially it is the carbohydrate consumption in the 24 h prior to sampling that impacts on serum B-OHB levels, rather than habitual carbohydrate intake.

While women were advised to fast for 9.5–12 h, the exact length of each woman’s fast prior to serum blood collection was not recorded. Serum B-OHB levels rise in pregnancy during fasting, with significant differences in serum B-OHB between pregnant and non-pregnant women first seen at 16 h into an overnight fast [6]. Potentially the variability in serum B-OHB levels in this study is due to differences in duration of fasting, rather than daily carbohydrate intake, although there was no correlation between B-OHB levels and timing of the start of the OGTT, which varied between 07:30 hours and 10:00 hours. Maternal serum B-OHB levels may also be affected by factors other than carbohydrate intake and glucose availability, including overall energy intake. A previous study has shown that maternal urinary ketone levels are associated with gut microbiome composition [11]. In particular, the genus *Roseburia* was more abundant in the gut microbiota of pregnant women with urinary ketones compared with women without urinary ketones. *Roseburia* is a butyrate-producing bacterium and studies have shown a link between butyrate and *Roseburia* in the gut and serum B-OHB levels [24]. This suggests that the microbiome may have a direct effect on serum B-OHB levels.

**Fig. 3:**
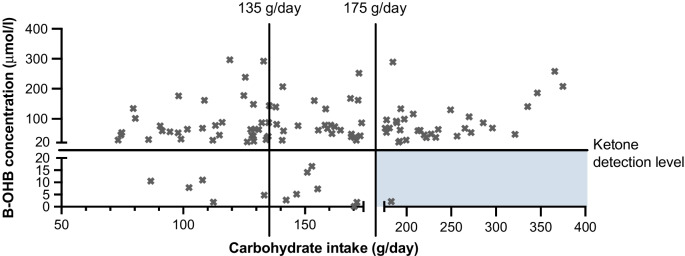
Representation of the B-OHB levels according to carbohydrate intake in the study cohort. The recommended daily carbohydrate intake of ≥175 g/day, covering 97.5% of the estimated daily needs of the maternal and fetal brain consumption, and the estimated mean requirements of 135 g/day, covering the maternal and fetal brain needs of 50% of the population, are indicated by the vertical bars. The shaded area represents the desired carbohydrate intake and B-OHB levels

Our finding that habitual daily carbohydrate intake is not correlated with fasting serum B-OHB levels in pregnancy is important. Current guidelines which recommend that women should consume a minimum of 175 g/day of carbohydrate are based on calculations rather than on clinical evidence. This is the first study to assess whether daily carbohydrate intake affects serum B-OHB levels. Our findings challenge the current dietary recommendations that women should consume a minimum amount of carbohydrate every day, since only a single participant of the 34 who consumed the recommended amount of carbohydrate had undetectable B-OHB levels whereas the other 33 had detectable levels (Fig. 3). Further research needs to be done to determine the main drivers of elevated ketone levels in pregnancy to ensure that clinicians can give the best possible dietary advice to patients. Future studies should recruit participants prospectively, include dietary data collection under supervised conditions using both food frequency questionnaires and 24 h dietary recall at multiple timepoints in pregnancy, together with accompanying measurements of circulating B-OHB levels. In addition, the exact duration of fasting should be recorded before the 75 g OGTT, preferably after consumption of a standardised meal on the evening before the test.

## Conclusion

Fasting serum B-OHB levels are not correlated with habitual daily carbohydrate intake at 28 weeks’ gestation in pregnant women with overweight and obesity. Further research is needed to further evaluate the relationship between carbohydrate intake and maternal ketone levels, as well as the main determinants of elevated ketone levels in pregnancy.

### Supplementary Information

Below is the link to the electronic supplementary material.

Supplementary file1 (PDF 123 KB)

## Data Availability

The data that support the findings of this study are not openly available due to reasons of sensitivity and are available from the corresponding author upon reasonable request. Data is located in controlled access data storage at The University of Queensland.

## Abbreviations

ACN: Acetonitrile
B-OHB: β-Hydroxybutyrate
DALI: The Diabetes and Pregnancy Vitamin D and Lifestyle Intervention for Gestational Diabetes Mellitus Prevention
2EBA: 2-Ethyl butyric acid
EDC: *N*-(3-Dimethylaminopropyl)-*N*′-ethyl carbodiimide
GDM: Gestational diabetes mellitus
HB: High B-OHB group
β-HBA: DL-β-hydroxybutyric acid sodium salt
HC: High-carbohydrate group
HDP: Hypertensive disorders of pregnancy
IQ: Intelligence quotient
LB: Low B-OHB group
LC: Low-carbohydrate group
LGA: Large for gestational age
3NPH: 3-Nitrophenyl hydrazine
SPRING: Study of Probiotic IN Gestational Diabetes
