# Supplementary material for: Habitual carbohydrate intake is not correlated with circulating β-hydroxybutyrate levels in pregnant women with overweight and obesity at 28 weeks' gestation
Source: Diabetologia. 2023 Nov 16;67(2):346–55. doi: 10.1007/s00125-023-06044-w (PMC10789657; doi:10.1007/s00125-023-06044-w)
Supplement: Supplementary file 1 — Supplementary file1 (PDF 123 KB) [file 125_2023_6044_MOESM1_ESM.pdf]

ESM table 1. Relative dietary intake across pregnancy until 28 weeks' gestation (mg nutrient/kJ)

	Low carbohydrate	High carbohydrate	P-value
N	53	53	
Carbohydrate (mg/kJ)	24.3 ±2.6	25.5 ± 2.8	0.02
Sugars (mg/kJ)	11.5 ± 2.7	12.1 ± 2.7	0.26
Starch (mg/kJ)	12.6 ± 2.3	13.3 ± 2.1	0.14
Fibre (mg/kJ)	2.8 ± 0.7	2.8 ± 0.6	0.54
Protein (mg/kJ)	11.6 ± 1.3	11.6 ± 1.5	0.99
Total fat (mg/kJ)	10.7 ± 1.0	10.2 ± 1.1	0.007
Saturated fat (mg/kJ)	4.7 ± 4.5	4.4 ± 0.6	0.01
Polyunsaturated fat (mg/kJ)	1.4 ± 0.5	1.3 ± 0.3	0.24
Monounsaturated fat (mg/kJ)	3.7 ± 0.4	3.6 ± 0.5	0.38

ESM table 2. Participant characteristics at 28 weeks' gestation grouped by B-OHB levels below (LB) or above (HB) the median of 65.4 µM.

	LB	HB	P-value
N	47	48	ND
Maternal age	32 (28-34)	31 (29-36)	0.56
Ethnicity Caucasian N (%)	43 (92)	46 (96)	0.44
Study allocation Probiotics/Placebo (N)	25 (53)/22 (47)	23 (48)/25 (48)	0.68
Parity < 1 (N (%))	13 (28)	6 (13)	0.08
Maternal BMI at 28 weeks (kg/m^2)	32.8 (30.2-39.7)	33.3 (30.0-38.0)	0.91
SBP (mmHg)	110 (105-115)	110 (104-120)	0.62
DBP (mmHg)	65 (60-70)	68 (60-72)	0.48
OGTT-Fasting (mmol/l)	4.2 (3.9-4.5)	4.2 (4.0-4.5)	0.72
OGTT-1 hour (mmol/l)	6.7 (5.7-8.0)	7.6 (6.8-8.1)	0.02
OGTT-2 hour (mmol/l)	6.0 (4.9-7.3)	6.3 (5.7-7.2)	0.20
Fasting insulin (pmol/l)	57.3 (36.5-91.5)	49.8 (34.5-69.0)	0.06
Fasting C-peptide (nmol/l)	0.8 (0.7-1.1)	0.7 (0.6-0.9)	0.07
Fasting cholesterol (mmol/l)	6.8 (5.7-7.8)	6.6 (6.1-7.4)	0.67
Fasting HDL cholesterol (mmol/l)	1.7 (1.5-2.0)	1.8 (1.5-2.1)	0.58
Fasting LDL cholesterol (mmol/l)	3.9 (3.1-4.6)	3.8 (3.3-4.5)	0.60
Fasting VLDL cholesterol (mmol/l)	1.0 (0.7-1.1)	0.9 (0.7-1.2)	0.63
Fasting triglycerides (mmol/l)	2.2 (1.6-2.5)	2.0 (1.6-2.6)	0.56
HOMA-IR	1.8 (1.1-2.9)	1.5 (1.0-2.1)	0.07
HOMA-B	276 (180-344)	223 (163-311)	0.22
GDM (N (%))	1 (2)	5 (10)	0.20
HDP (N)	2 (4)	6 (13)	0.27
GWG (kg)*	7.2 (4.4-11.2)	9.0 (5.0-13.0)	0.23

Gestational Age at delivery (weeks)	40.1 (39.3-40.9)	39.4 (38.7-40.8)	0.10
Infant gender Female (N(%))/Male (N(%))	22 (47)/25 (53)	27 (56)/21(44)	0.41
Infant birth weight (g)	3686 (3464-4013)	3670 (3391-3984)	0.83
SGA (<10th centile) (N)	0	1	0.99
LGA (>90th centile) (N)	6	11	0.42
Infant length (cm)	53.0 (50.8-54.0)	52.5 (50.0-54.0)	0.94
Infant head circumference (cm)	35.0 (34.0-36.0)	36.0 (34.0-36.5)	0.20

DBP, diastolic blood pressure; GDM, gestational diabetes mellitus; GWG, gestational weight gain; HB, high B-OHB; HDP, hypertensive disorders of pregnancy; HOMA-B, homeostatic assessment of b-cell function; HOMA-IR, homeostatic assessment of insulin resistance; LB, low B-OHB ; LGA, large for gestational age; N, number; OGTT, oral glucose tolerance test; SBP, systematic blood pressure; SGA, small for gestational age; *, across gestation. Data displayed as median (IQR)

ESM table 3. Dietary intake in pregnancy until 28 weeks' gestation grouped by B-OHB levels below (LB) or above (HB) the median of 65.4 μM.

	LB	HB	P-value
N	46*	46	ND
Overall Energy (kJ/d)	5979 (4743-8238)	6323 (4974-7511)	0.34
Carbohydrate (g/d)	148 (123-195)	160 (130-193)	0.34
Sugars (g/d)	71 (51-100)	74 (57-99)	0.53
Starch (g/d)	79 (59-99)	84 (68-115)	0.27
Fibre (g/d)	16 (12-23)	18 (14-22)	0.27
Protein (g/d)	71 (54-103)	72 (53-94)	0.90
Total fat (g/d)	61 (46-88)	68 (51-84)	0.29
Saturated fat (g/d)	27 (21-35)	30 (25-74)	0.26
Polyunsaturated fat (g/d)	8 (6-10)	8 (6-11)	0.5
Monounsaturated fat (g/d)	21 (16-31)	23 (17-30)	0.34

N, number; ND, not determined; *, no dietary data available from 1 participant in the LB group
Data presented as median (IQR)

ESM table 4. Participant characteristics at 28-weeks gestation and correlation with B-OHB levels in all participants (normoglycaemic and with GDM)

	Spearman's rho (95%CI)	P-value
Maternal age	0.09 (-0.12 – 0.29)	0.37
Maternal BMI at 28 weeks (kg/m^2)	0.06 (-0.15 – 0.27)	0.56
SBP (mmHg)	0.14 (-0.07 – 0.34)	0.17
DBP (mmHg)	0.13 (-0.08 – 0.33)	0.21
OGTT-Fasting (mmol/l)	0.12 (-0.09 – 0.32)	0.25
OGTT-1 hour (mmol/l)	0.25 (0.04 – 0.43)	0.02
OGTT-2 hour (mmol/l)	0.12 (-0.09 – 0.32)	0.27
Fasting insulin (mU/L)	-0.15 (-0.35 – 0.07)	0.16
Fasting cholesterol (mmol/l)	-0.03 (-0.18 – 0.24)	0.75
Fasting HDL cholesterol (mmol/l)	0.03 (-0.18 – 0.24)	0.76
Fasting LDL cholesterol (mmol/l)	0.04 (-0.17 – 0.24)	0.72
Fasting VLDL cholesterol (mmol/l)	0.02 (-0.23 – 0.19)	0.82
Fasting triglycerides (mmol/l)	-0.03 (-0.24 – 0.18)	0.77
HOMA-IR	-0.15 (-0.35 – 0.07)	0.17
HOMA-B	-0.05 (-0.26 – 0.17)	0.64
GWG (kg)*	0.09 (-0.12 – 0.29)	0.39
Infant birth weight (g)	-0.06 (-0.26 – 0.15)	0.59
Infant birth length (cm)	-0.03 (-0.25– 0.18)	0.76
Infant head circumference (cm)	0.06 (-0.16 – 0.27)	0.60

DBP, diastolic blood pressure; GDM, gestational diabetes mellitus; GWG, gestational weight gain; HB, high B-OHB; HDP, hypertensive disorders of pregnancy; HOMA-B, homeostatic assessment of b-cell function; HOMA-IR, homeostatic assessment of insulin resistance; LB, low B-OHB; OGTT, oral glucose tolerance test; SBP, systolic blood pressure; *, across gestation

ESM table 5. Dietary intake in pregnancy until 28 weeks gestation and correlation with B-OHB levels in all participants (normoglycaemic and with GDM)

	Spearman's rho (95%CI)	P-value
Overall Energy (kJ/d)	0.09 (-0.12 – 0.29)	0.40
Carbohydrate (g/d)	0.09 (-0.07 – 0.31)	0.40
Carbohydrate (mg/kJ)	0.03 (-0.18 – 0.24)	0.75
Sugars (g/d)	0.03 (-0.18 – 0.23)	0.80
Starch (g/d)	0.12 (-0.09 – 0.32)	0.24
Fibre (g/d)	0.14 (-0.06 – 0.35)	0.17
Protein (g/d)	0.005 (-0.20 – 0.21)	0.96
Total fat (g/d)	0.12 (-0.09 – 0.31)	0.27
Saturated fat (g/d)	0.12 (-0.09 – 0.32)	0.23
Polyunsaturated fat (g/d)	0.09 (-0.12 – 0.29)	0.38
Monounsaturated fat (g/d)	0.11(-0.10 – 0.31)	0.31
